# P-353. Ten Years Results on the Effectiveness and Safety of DTG+3TC as a Switch Regimen in a Multicenter Cohort

**DOI:** 10.1093/ofid/ofaf695.571

**Published:** 2026-01-11

**Authors:** Gianmaria Baldin, Arturo Ciccullo, Adriana Cervo, Letizia Oreni, Maria Mazzitelli, Filippo Lagi, Marianna Menozzi, Alberto Borghetti, Francesca Lombardi, Andrea Giacomelli, Massimiliano Fabbiani, Stefano Rusconi, Annamaria Cattelan, Spinello Antinori, Cristina Mussini, Simona Di Giambenedetto

**Affiliations:** Fondazione Policlinico A. Gemelli IRCCS, Roma, Lazio, Italy; Infectious Diseases Unit, Ospedale San Salvatore, L’Aquila, Italy, L'Aquila, Abruzzi, Italy; University Hospital Policlinic of Modena, Modena, Emilia-Romagna, Italy; III Infectious Diseases Unit, ASST Fatebenefratelli Sacco, Luigi Sacco Hospital, Milan, Italy., Milan, Lombardia, Italy; Infectious and Tropical Diseases Unit, Padua University Hospital, Padua, Italy., Padova, Veneto, Italy; Department of Experimental and Clinical Medicine, University of Florence, Florence, Italy., Firenze, Toscana, Italy; Department of Infectious Diseases, Azienda Ospedaliero-Universitaria of Modena, Modena, Italy, Modena, Emilia-Romagna, Italy; Infectious Diseases Unit, Department of Clinical and Experimental Medicine, Azienda Ospedaliera Universitaria Pisana, Pisa, Italy., Pisa, Toscana, Italy; Fondazione Policlinico A. Gemelli IRCCS, Roma, Lazio, Italy; III Infectious Diseases Unit, ASST Fatebenefratelli Sacco, Luigi Sacco Hospital, Milan, Italy., Milan, Lombardia, Italy; Department of Medical Sciences, Infectious and Tropical Diseases Unit, University Hospital of Siena, Siena, Italy, Siena, Toscana, Italy; Ospedale Civile di Legnano ASST Ovest Milanese, University of Milan, Legnano, Italy., Legnano, Lombardia, Italy; Infectious and Tropical Diseases Unit, Padua University Hospital, Padua, Italy., Padova, Veneto, Italy; III Infectious Diseases Unit, ASST Fatebenefratelli Sacco, Luigi Sacco Hospital, Milan, Italy., Milan, Lombardia, Italy; Infectious Disease Clinic, Azienda Ospedaliero-Universitaria di Modena; University of Modena and Reggio Emilia, Modena, Italy., MODENA, Emilia-Romagna, Italy; Infectious Diseases Unit, Fondazione Policlinico Universitario Agostino Gemelli IRCCS, Rome, Italy, Rome, Lazio, Italy

## Abstract

**Background:**

We aim to assess the efficacy, safety and tolerability of DTG+3TC as a switch regimen after 10 years of its introduction in clinical practice.

**Figure:**
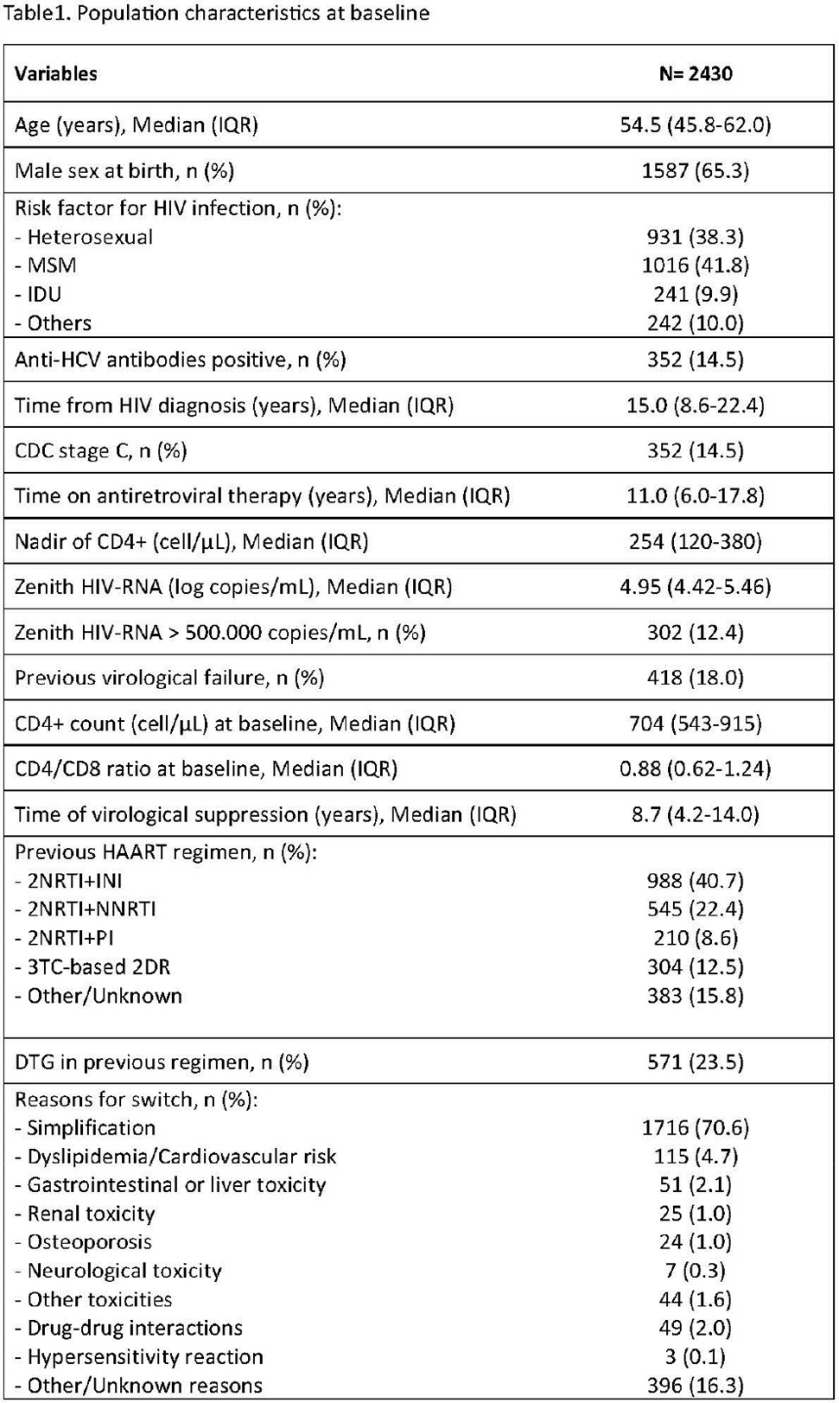

**Methods:**

An observational study was conducted with virologically suppressed PLWH switching to DTG+3TC in a multicenter cohort. Exclusion criteria included HBsAg+ status and M184V resistance mutation. We employed Kaplan-Meyer survival analysis for virological failure (VF) and treatment discontinuation (TD), Cox-regression for VF or TD predictors, and linear mixed models for immunological and metabolic parameter changes.

**Results:**

We enrolled 2430 PLWH: 1587 (65.3%) were males, with a median age of 54.5 yrs. Full population characteristics are shown in Table1.

During 9199 PYFU, we observed 82 VF (0.9 per 100 PYFU); no emergent resistance mutations were registered among VF. Estimated probabilities of maintaining virological suppression (VS) at 240 and 480 wks were 96.2% and 92.8%, respectively. At a multivariate analysis, a history of at least one previous VF before switch (vs no event, aHR 2.09, p=0.005) and a time of VS < 8 years before switch (vs VS > 8 years, aHR 1.82, p=0.021) were significantly associated with a higher risk of VF.

During 9287 PYFU, we observed 339 TD (3.7 per 100 PYFU). Estimated probabilities of maintaining DTG/3TC were 83.8% at 240 wks and 79.4% at 480 wks. Discontinuations for toxicity were 78 (23.0% of all TD), with CNS toxicity accounting for 25 cases. Other frequent reasons for TD were: switch to CAB+RPV (64, 18.9%) and treatment intensification (32, 9.4%).

At a multivariate analysis, we found that a HIV-RNA peak > 500.000 cps/mL (aHR 1.55, p=0.006) and the presence of HBcAb (aHR 1.60, p=0.002) were significantly associated with a higher risk of TD; meanwhile, the presence of DTG in the ARV regimen before switch, (aHR 0.51, p=0.001) was inversely correlated with TD risk.

After 240 weeks, we registered a significant improvement in both CD4+ count (+59, p < 0.001) and CD4/CD8 ratio (+0.1, p < 0.001).

As to metabolic parameters, we observed significant improvement in total cholesterol, tryglicerides and HDL cholesterol at both 240 and 480 weeks (p < 0.02 for all).

**Conclusion:**

We assessed the efficacy and safety of DTG+3TC in the long term while also showing neutral impact on metabolic parameters.

**Disclosures:**

All Authors: No reported disclosures

